# Milk Fat Globule-Epidermal Growth Factor-8 Pretreatment Attenuates Apoptosis and Inflammation *via* the Integrin-β3 Pathway after Surgical Brain Injury in Rats

**DOI:** 10.3389/fneur.2018.00096

**Published:** 2018-02-26

**Authors:** Yicai Xiao, Gaofeng Li, Yujie Chen, Yuchun Zuo, Kauthar Rashid, Tibiao He, Hua Feng, John H. Zhang, Fei Liu

**Affiliations:** ^1^Department of Neurosurgery, The Third Xiangya Hospital, Central South University, Hunan, Changsha, China; ^2^Departments of Oncology, Zhuzhou Central Hospital, Hunan, Zhuzhou, China; ^3^Department of Neurosurgery, Southwest Hospital, Third Military Medical University, Chongqing, China; ^4^Neuroscience Research Center, Loma Linda University, Loma Linda, CA, United States

**Keywords:** milk fat globule-epidermal growth factor-8, surgical brain injury, apoptosis, inflammation, integrin β3

## Abstract

Iatrogenic brain injury inevitably occurs in neurosurgical operations, leading to brain edema, ischemia, intracranial hematoma, and other postoperative complications, eventually worsening neurological outcomes of patients. If apoptotic cells are not rapidly eliminated by phagocytic engulfment, they may communicate with surrounding cells to undergo secondary necrosis and releasing toxic signals. Recent studies have shown that milk fat globule-epidermal growth factor-8 (MFGE8), which promotes phagocytosis and inhibits inflammation, is an endogenous protective factor in response to brain infarction, Alzheimer’s disease, subarachnoid hemorrhage, and prion disease. In the present study, we sought to investigate the different effects of both pretreated and posttreated recombinant milk fat globule-epidermal growth factor-8 (rhMFGE8) for the surgical brain injury (SBI) rat model and potential involvement of its receptor integrin β3 for apoptosis and neuroinflammation after SBI. One hundred and sixty-seven male rats were employed in the preset study. Experiment 1 was performed to evaluate neurological scores and MFGE8, cleaved caspase-3 (CC3), and interleukine-1 beta (IL-1β) levels at 3, 24, and 120 h after SBI. Experiment 2 was performed to evaluate the effects of rhMFGE8 pretreatment (10 min before SBI) and rhMFGE8 posttreatment (6 h after SBI) on brain edema at 24 and 72 h after SBI. Experiment 3 was performed to evaluate the potential anti-apoptotic and anti-inflammatory effects of rhMFGE8 pretreatment and posttreatment. Experiment 4 sought to investigate the involvement of the integrin-β3 signal in the effects of MFGE8 pretreatment. Our data showed rhMFGE8 pretreatment alleviated neurological deficits and decreased brain water content and apoptotic cells in the SBI model, which exhibited neurological dysfunction, apoptosis, and inflammation. Meanwhile, MFGE8 siRNA, which inhibited endogenous MFGE8 expression, significantly increased IL-1β, TUNEL positive cells, and CC3. Furthermore, knockdown of its receptor integrin β3 by siRNA abolished the effects of rhMFGE8 in the SBI model. In conclusion, we found that rhMFGE8 pretreatment effectively alleviated neurological deficits and decreased brain water content and apoptotic cells in the SBI model through the MFGE8/integrin-β3 pathway, and treatment time was an important factor in achieving curative effects. Therefore, MFGE8 pretreatment may serve as a promising therapeutic strategy for SBI patients.

## Introduction

Iatrogenic brain injury inevitably occurs in neurosurgical operations, leading to brain edema, ischemia, intracranial hematoma, and other postoperative complications, eventually worsening neurological outcomes of patients (1). The mechanisms of this damage include cortical incision, electrocauterization, retraction, and vascular or microvascular occlusion. A surgical brain injury (SBI) rodent model has been developed and demonstrated brain edema, cell apoptosis, inflammation, and oxidative stress in the peri-resection area (2–6). Among these pathophysiologies, inflammation plays a pivotal role in the secondary brain injuries due to different etiologies, including but not limited to trauma, ischemia, neurodegeneration, and excitotoxicity (7–9). If apoptotic cells are not rapidly eliminated by phagocytic engulfment, they may communicate with surrounding cells to undergo secondary necrosis and releasing toxic signals, such as the inflammatory factor interleukine-1 beta (IL-1β) (10–12). Therefore, timely apoptotic cell clearance could alleviate inflammatory damage.

Milk fat globule-epidermal growth factor-8 (MFGE8, also called lactadherin), a multifunctional glycoprotein which was originally understood as part of the milk fat globule membrane (13–15), is instrumental in cell–cell interactions and diverse physiological and pathophysiological functions, including fertilization, angiogenesis, and phagocytosis of apoptotic cells (16). Recent studies have shown that MFGE8, which promotes phagocytosis and inhibits inflammation, is an endogenous protective factor in response to brain infarction (17, 18), Alzheimer’s disease (19, 20), subarachnoid hemorrhage (SAH) (16), and prion disease (21). However, Kinugawa et al. reported that MFGE8 does not orchestrate the apoptotic neurons clearance in a Parkinson’s disease mouse model (22). Moreover, Neher et al. found that MFGE8^−/−^ mice had therapeutic targets even with a lack of MFGE8 expression in transient cerebral ischemia (23). MFGE8 thus has dual effects in different models.

To date, the effects of MFGE8 in the SBI model are unclear. Moreover, SBI patients show similarities to the SBI model after pretreatment with drugs, in contrast to stroke and trauma patients. Therefore, in the present study, we sought to investigate the different effects of both pretreated- and posttreated recombinant milk fat globule-epidermal growth factor-8 (rhMFGE8) for SBI rat model and potential involvement of its receptor integrin β3 for apoptosis and neuroinflammation after SBI. The results preliminarily demonstrated that MFGE8 is a promising therapeutic target for SBI and that treatment time was an important factor in achieving a curative effect.

## Materials and Methods

### Experimental Design

One hundred and sixty-eight male Sprague-Dawley rats, age between 8 and 12 weeks, weighing 280–350 g, were employed in the present study. Rats were housed in a humidity and temperature-controlled room with food and water *ad libitum*. The light was controlled in a 12-h light/dark cycle. These rats were acclimatized for more than 3 days before surgical procedures. All experimental protocols were approved by the Ethics Committee of Central South University and performed according to the eighth edition of the National Institutes of Health Guide for the Care and Use of Laboratory Animals and reported according to the ARRIVE guideline.

**Figure 1:**
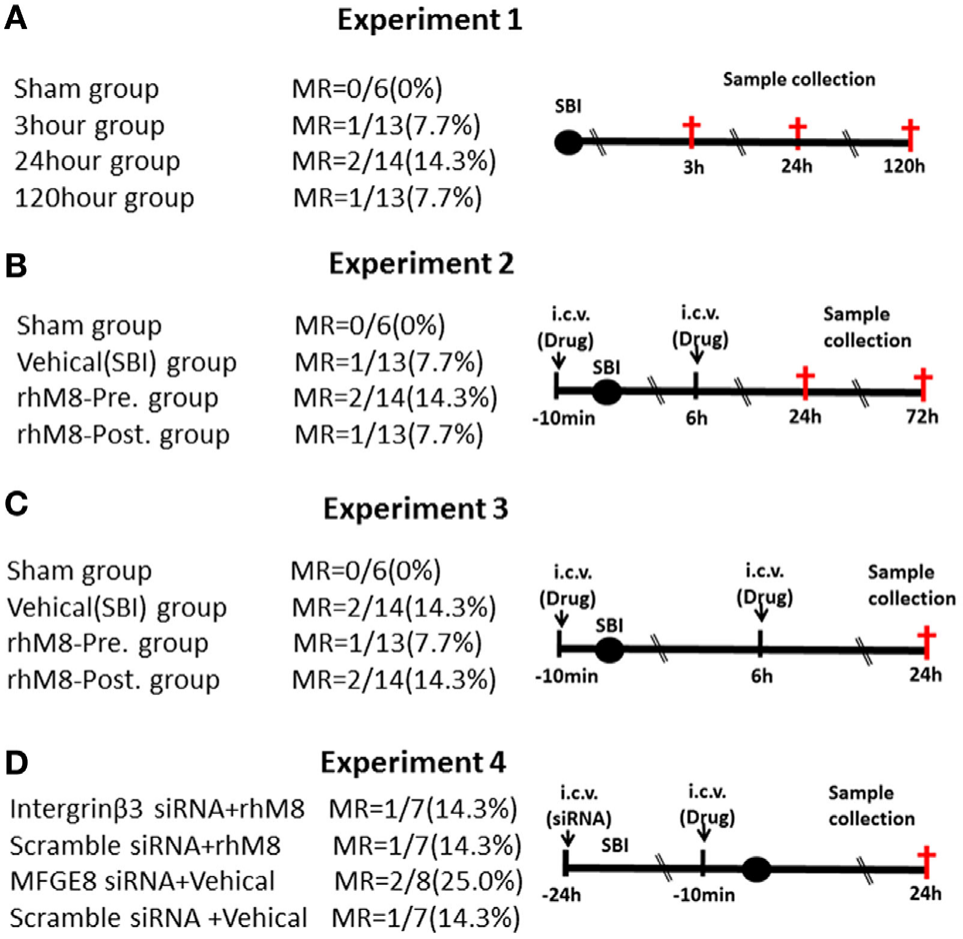
Experimental design and groups. There are no significant differences of mortality rate (MR) among different surgical brain injury (SBI) groups **(A–D)**. In experiment 3 **(C)**, each surgery group divided into two subgroups: one for western blot (the sham subgroup share with experiment 1) and the other for immunohistochemistry. It was recombinant milk fat globule-EGF factor-8 (rhMFGE8) pretreatment group (rhM8-pre) that rhMFGE8 was injected by i.c.v. at 10 min before SBI, and rhMFGE8 posttreatment group (rhM8-post) that rhMFGE8 was injected by i.c.v. at 6 h after SBI.

Experiments designs are illustrated in Figure 1. Experiment 1 (Figure 1A) was performed to evaluate neurological scores (*n* = 6 at each timepoint) and MFGE8, cleaved caspase-3 (CC3), and IL-1β levels (*n* = 6 at each timepoint) at 3, 24, and 120 h after SBI. Experiment 2 (Figure 1B) was performed to evaluate the effects of rhMFGE8 pretreatment (10 min before SBI) and rhMFGE8 posttreatment (6 h after SBI) on brain edema (*n* = 6 at each timepoint) at 24 and 72 h after SBI. Experiment 3 (Figure 1C) was performed to evaluate the potential anti-apoptotic and anti-inflammatory effects of rhMFGE8 pretreatment and posttreatment (*n* = 6). Experiment 4 (Figure 1D) sought to investigate the involvement of the integrin-β3 signal in the effects of MFGE8 pretreatment (*n* = 6 for each group).

### Surgical Brain Injury

As previously described (6), the SBI model was performed by partially resecting the right frontal lobe of rat brain. Briefly, rats were intraperitoneally anesthetized with 50-mg/kg sodium pentobarbital, followed with 0.5-mg/kg atropine intraperitoneally to inhibit respiratory secretions. Under a surgical dissecting microscope, a midline incision was made in scalp, and a 4-mm edge square cranial window, beginning at the bregma at right frontal skull, was removed using a bone drill. Then, the dura was excised. A partial right frontal lobectomy was made through two separate incisions leading away from the bregma along the sagittal and coronal, approximately 2-mm lateral to the sagittal suture and 1-mm proximal to the coronal suture. Once the brain tissue was excised, electrocautery was applied briefly (1–2 s total) to the cut surfaces used for hemostasis. Sham animals underwent only a craniotomy.

### Intracerebroventricular Infusion

Intracerebroventricular infusion was performed as previously described (24). Briefly, rats were placed in a stereotactic apparatus after they were anesthetized with sodium pentobarbital. Then, the skin was incised, and a burr hole was drilled at the following coordinates relative to the bregma: 1.5-mm posterior and 1.0-mm lateral (left). The needle of a 10-µL Hamilton syringe (Microliter 701; Hamilton Company, Reno, NV, USA) was inserted below the horizontal plane of the skull. Drugs, which included rhMFGE8 (Sigma, 3.3 μg/3 μL), MFGE8 siRNA, integrin-β3 siRNA, or scrambled siRNA (500 pmol/3 μL, Santa Cruz Biotechnology), were injected at a rate of 0.5 µL/min by a pump on schedule according to the design.

### Neurobehavioral Test

Neurobehavioral scores included modified Garcia scoring test and beam balance test at 1 h before euthanasia (25). Tests were performed by a blinded observer. The modified Garcia test score ranges from 0 to 18, which was assigned based on spontaneous activity, spontaneous movement of all limbs, fore paw outstretching, climbing, body proprioception, and response to whisker stimulation. The beam balance test (0–4) was based on the walking distance on a 45-cm long and 22.5-mm diameter wooden beam between two platforms. Briefly, rats were placed perpendicularly on the midpoint of the beam and allowed to traverse the beam for 60 s. 0 point for “Does not move and falls off the rod within 30 s,” 1 point for “Does not move but stays on the rod for more than 30 s (in any manner),” 2 point for “Moves less than half the distance to the platform (22.5 cm) in ≤60 s,” 3 point for “Moves at least half the distance to the platform (22.5 cm) in ≤60 s,” and 4 point for “Reaches the platform or moves at least 45 cm in ≤60 s.”

### Brain Water Content

Rat brains were removed under anesthesia at 24 or 72 h after surgery. Brains were quickly separated into six parts: right/left frontal, right/parietal, cerebellum, and brain stem. The coronal middle line was defined as the cut-off for the frontal lobe and parietal lobe. Samples were immediately weighed on a high-precision balance (wet weight) and after drying in an oven at 105°C at 48 h later (dry weight). The percentage of brain water content was calculated as (wet weight−dry weight)/wet weight × 100%.

### Western Blot Analysis

The right frontal tissues were collected 24 h after SBI. Samples were snap-frozen in liquid nitrogen and stored at 80°C until use. Western blot was performed as described previously (25). Primary antibodies against MFGE8, β-Actin (1:4,000, Santa Cruz Biotechnology, Santa Cruz, CA, USA), IL-1β (1:2,000, Abcam, Cambridge, MA, USA), and CC3 (1:2,000, Cell Signaling Technology, Danvers, MA, USA) were used.

### Immunohistochemistry

Animals were euthanized at 24 h under deep anesthesia. Brain tissue was fixed with sucrose and formalin. Then, 10-µm coronal sections were cut in a cryostat. As described previously (24), immunofluorescence staining was performed by using an *in situ* cell death detection kit (Roche, Indianapolis, IN, USA) as the manufacturer’s instruction. Four views/pictures were taken along the periphery of surgical resection and calculated for each rat. The TUNEL-positive cells were quantified at ×200 magnification in a blinded manner by other researchers and expressed as cells/mm^2^.

### Statistical Analysis

Statistical analysis was performed using GraphPad Prism 5 (GraphPad Software Inc., San Diego, CA, USA). Quantitative data were presented as the mean ± SEM. One-way analysis of variance (ANOVA) followed by Tukey’s multiple comparisons test was used for different groups’ comparison. Chi-square tests were used for behavior score analyses. *P* < 0.05 was considered statistically significant.

## Results

All sham-operated rats survived. A total of 168 male rats underwent SBI; 18 rats died after SBI, and the mortality of SBI rats was 10.7%. But the mortality did not exhibit significantly different among the experimental groups (Figure 1, *P* > 0.05).

### MFGE8, CC3, and IL-1β Expression Increased after SBI

**Figure 2:**
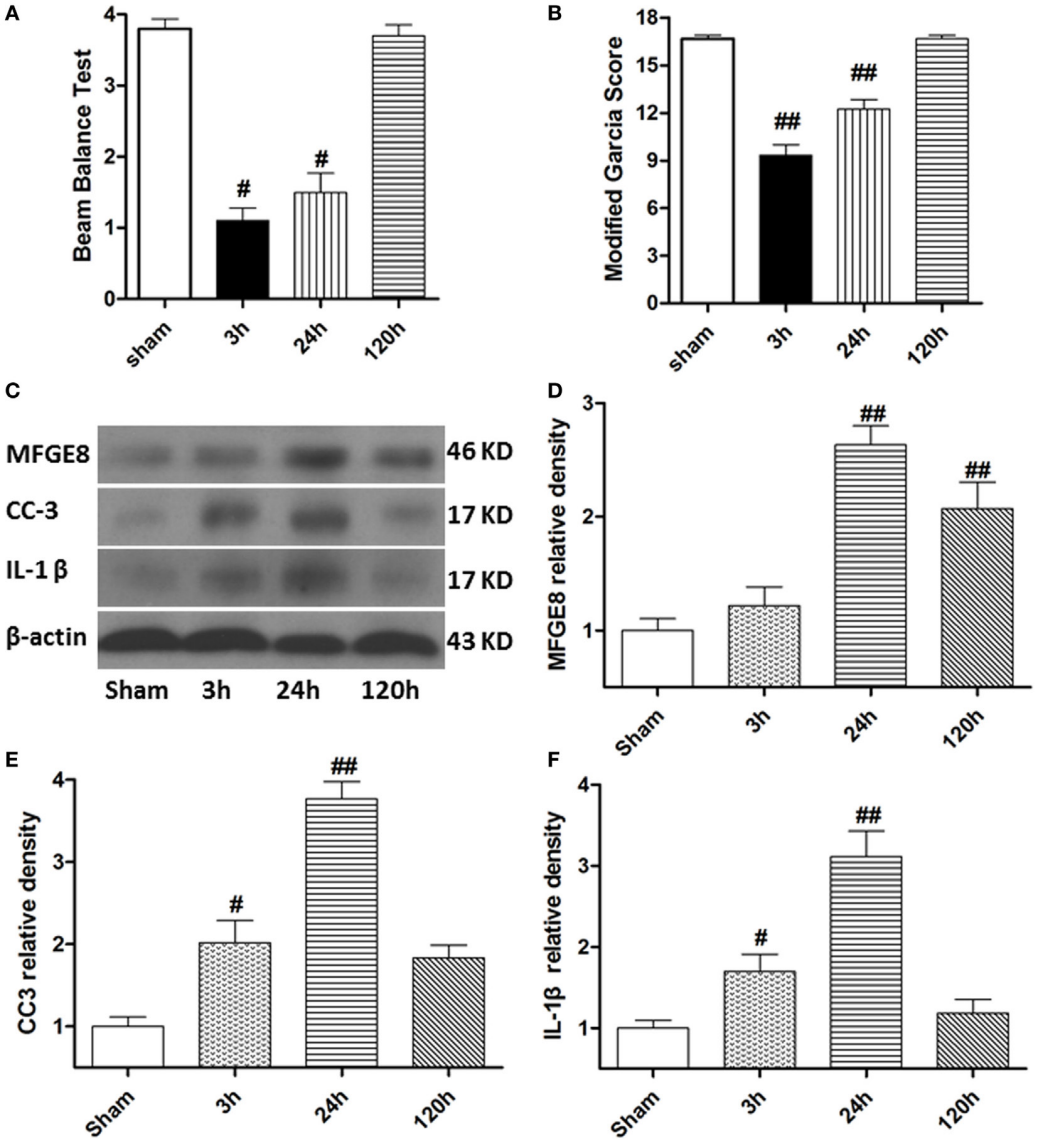
Time course of neurological scores, and expression of milk fat globule-EGF factor-8 (MFGE8), cleaved caspase-3 (CC3), and interleukine-1 beta (IL-1β) after surgical brain injury (SBI). **(A,B)** Animals showed severely neurological deficits from 3 h after subarachnoid hemorrhage, improved as time passed (at 120 h after SBI), and was not significant difference with sham. Representative blots **(C)** and quantitative analysis of MFGE8, CC3, and IL-1β **(D–F)** show the levels of MFGE8, CC3, and IL-1β all increased after SBI and peaked at 24 h, but expression of MFGE8 increasing **(D)** was not significant at 3 h after SBI. *n* = 6 for each time point. ^#^*P* < 0.05 vs. sham group and ^##^*P* < 0.01 vs. sham group.

At 3 h after SBI, the rat models exhibited obvious neurological deficits in the modified Garcia score (Figure 2A, *P* < 0.05) and beam balance score (Figures 2B, *P* < 0.05) comparing to sham rats, and these differences further increased at 24 h after SBI (*P* < 0.05). However, compared with sham rats, the SBI rats showed no significant differences in neurological function on the modified Garcia score and beam balance score at 120 h after SBI (Figures 2A,B, *P* > 0.05). Western blots demonstrated that MFGE8, CC3, and IL-1β were all poorly expressed in sham rats (Figures 2C–F). They all increased from 3 h after SBI, with a peak at 24 h, which lasted 120 h after SBI (Figures 2C–F, *P* < 0.01). The increase in CC3 and IL-1β was significant at 3 h after SBI compared with that of sham rats (Figures 2E,F, *P* < 0.05), but MFGE8 expression was not significantly altered (Figure 2D, *P* > 0.05). However, MFGE8 was still significantly higher than that in the sham group at 120 h after SBI (*P* < 0.01), but CC3 and IL-1β showed no differences compared with the expression in sham rats (Figures 2C–F, *P* > 0.05).

### Administration of rhMFGE8 Alleviated Neurological Deficits and Decreased Brain Water Content in SBI Rats

**Figure 3:**
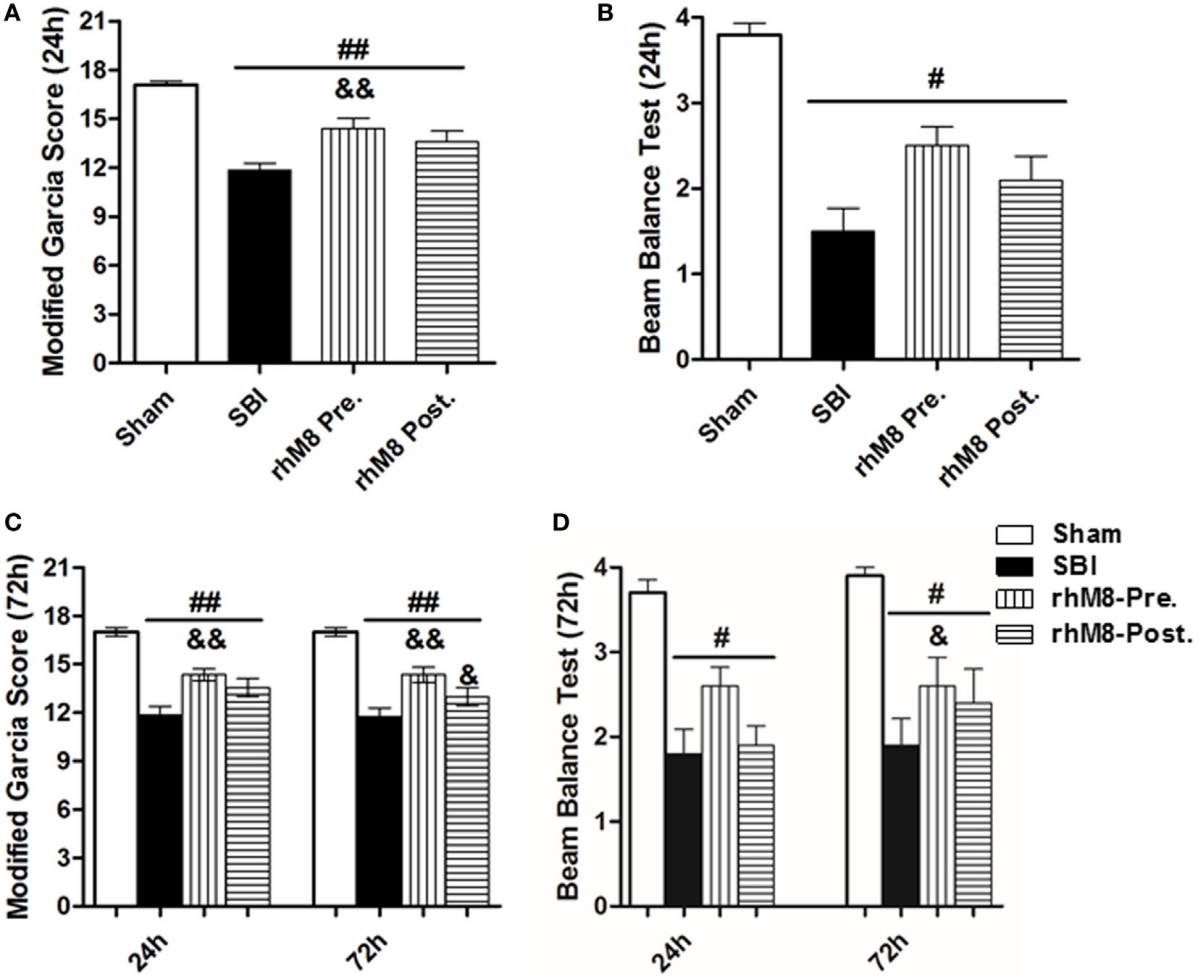
The different effect between recombinant milk fat globule-EGF factor-8 (rhMFGE8) pretreatment (rhM8-pre) and rhMFGE8 posttreatment (rhM8-post) on neurological scores at 24 and 72 h after surgical brain injury (SBI). **(A,B)** rhM8-pre significantly improved the modified Garcia neurological function at 24 h. However, rhM8-post was not significant. **(C,D)** At 72 h, rhM8-pre significantly improved the modified Garcia neurological function and balance function, but rhM8-pre only significantly improved the modified Garcia neurological function. *n* = 6 for each group ^#^*P* < 0.05 vs. sham group, ^##^*P* < 0.01 vs. sham group, ^&^*P* < 0.05 vs. SBI group, and ^&&^*P* < 0.01 vs. SBI group.

**Figure 4:**
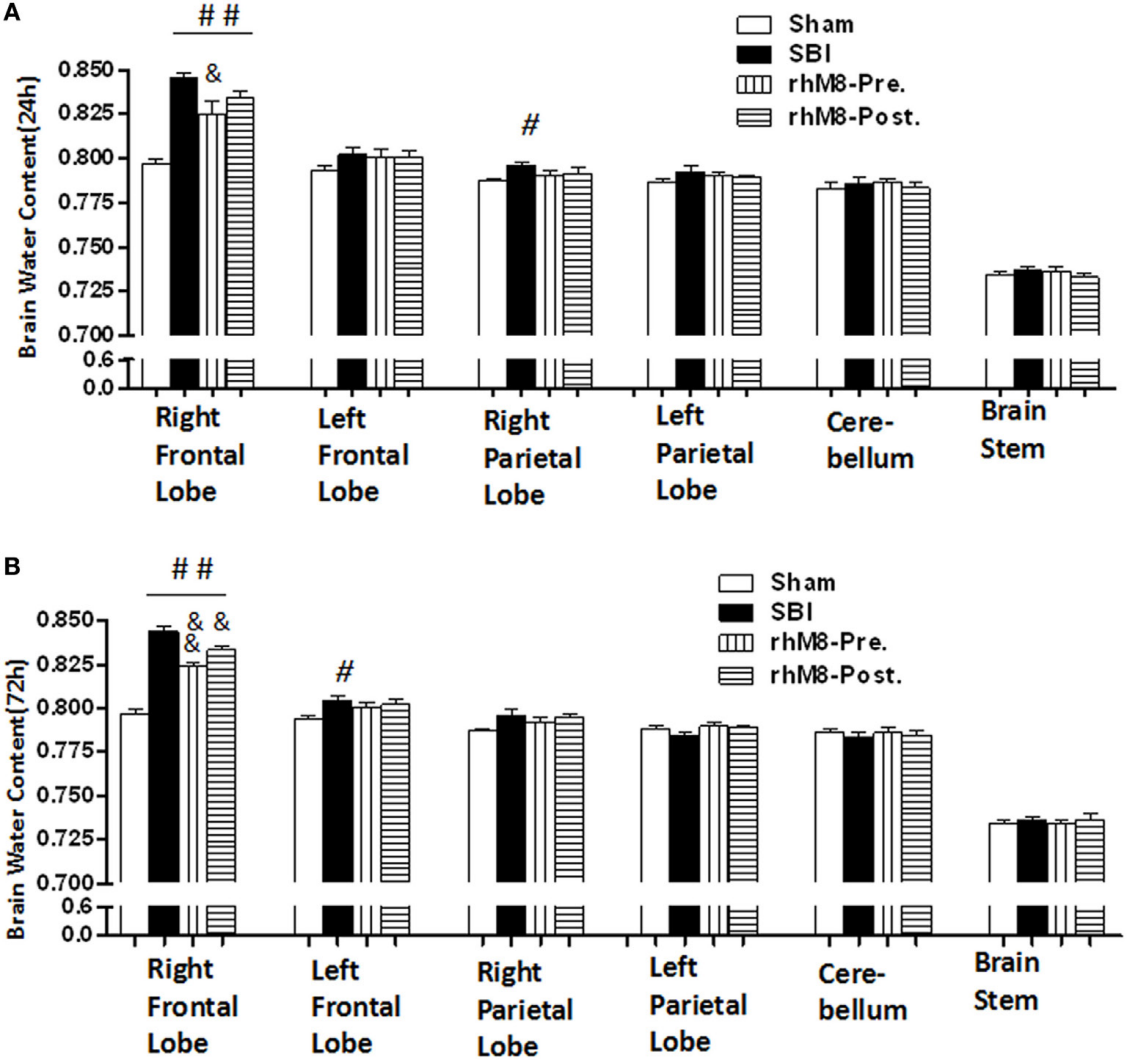
The different effects between recombinant milk fat globule-EGF factor-8 (rhMFGE8) pretreatment (rhM8-pre) and rhMFGE8 posttreatment (rhM8-post) on brain edema at 24 and 72 h. **(A,B)** rhM8-pre significantly reduced brain water content in right (ipsilateral) frontal lobes at 24 and 72 h. However, the decreasing of brain water content in rhM8-post was not significant. *n* = 6 for each group. ^#^*P* < 0.05 vs. sham group, ^##^*P* < 0.01 vs. sham group, ^&^*P* < 0.05 vs. surgical brain injury (SBI) group, and ^&&^*P* < 0.01 vs. SBI group.

There was a significant decrease in the modified Garcia score (Figures 3A,C *P* < 0.01) and the beam balance score (Figures 3B,D, *P* < 0.05) and aggravated brain water content in the right frontal lobe (ipsilateral) (Figures 4A,B, *P* < 0.01) in all rats subjected to SBI, with or without treatment, at 24 and 72 h compared with rats in the sham group.

Recombinant milk fat globule-epidermal growth factor-8 was administered intracerebroventricularly at two time points: 10 min before SBI (rhMFGE8 pretreatment) and 6 h after SBI (rhMFGE8 posttreatment). rhMFGE8 pretreatment significantly improved the modified Garcia score at 24 h (Figure 3A, *P* < 0.01) and 72 h (Figure 3C, *P* < 0.05) and increased the beam balance score only at 72 h (Figure 3D, *P* < 0.05). Moreover, rhMFGE8 pretreatment significantly decreased the brain water content at 24 h (Figure 4A, *P* < 0.05) and 72 h (Figure 4B, *P* < 0.01). However, posttreatment only increased the modified Garcia score (Figure 3C, *P* < 0.05) and decreased the brain water content at 72 h (Figure 4B, *P* < 0.01).

### Administration of rhMFGE8 Decreased TUNEL-Positive Cells 24 h after SBI, and Pretreatment Was Effective

**Figure 5:**
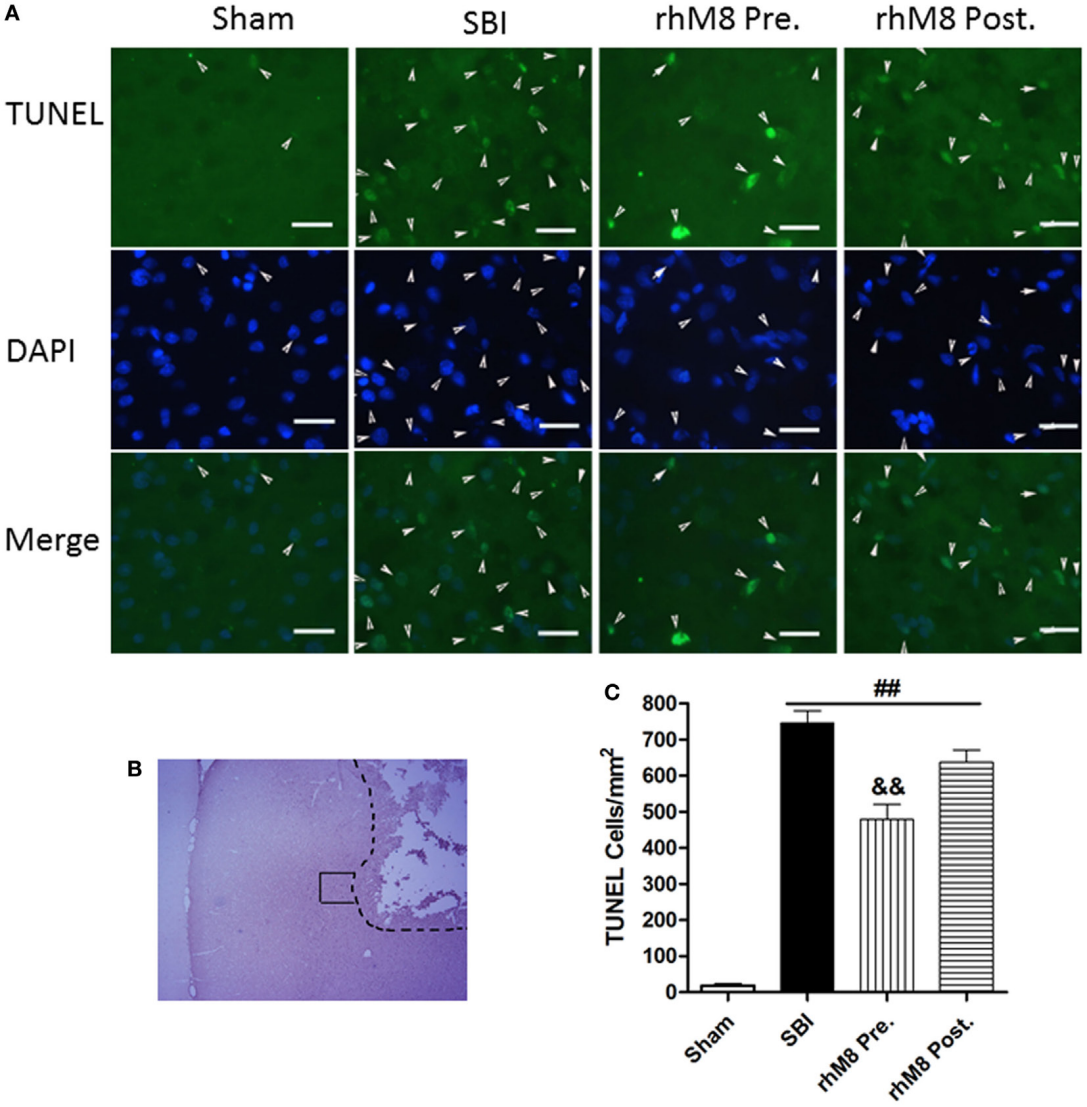
The different effects between recombinant milk fat globule-EGF factor-8 (rhMFGE8) pretreatment (rhM8-pre) and rhMFGE8 posttreatment (rhM8-post) on TUNEL-positive cells at 24 h after surgical brain injury (SBI). **(A)** Representative microphotographs showed the colocalization of TUNEL (green)-positive with DAPI (blue) cells along the periphery of surgical resection **(B)** at 24 h after SBI. **(C)** Semi-quantitative analysis of TUNEL-positive cells showed that the number of apoptotic cells was significantly increased after SBI and rhMFGE8 significantly decreased the number of apoptotic cells, but rhM8-pre had better effective. Scale bar = 50 µm; *n* = 6 for each group. ^##^*P* < 0.01 vs. sham group, ^&^*P* < 0.05 vs. SBI group, and ^&&^*P* < 0.01 vs. SBI group.

TUNEL staining in the right frontal lobe indicated that the sham group had few TUNEL-positive cells (Figure 5). Stained cells increased substantially along the periphery of surgical resection in the SBI model. Administration of rhMFGE8 decreased the abundance of positive cells (Figure 5A), but only pretreatment had significant effects (Figures 5B,C, *P* < 0.01).

### Administration of rhMFGE8 Decreased Expression of CC3 and IL-1β at 24 h after SBI, and Pretreatment Was More Effective than Posttreatment

**Figure 6:**
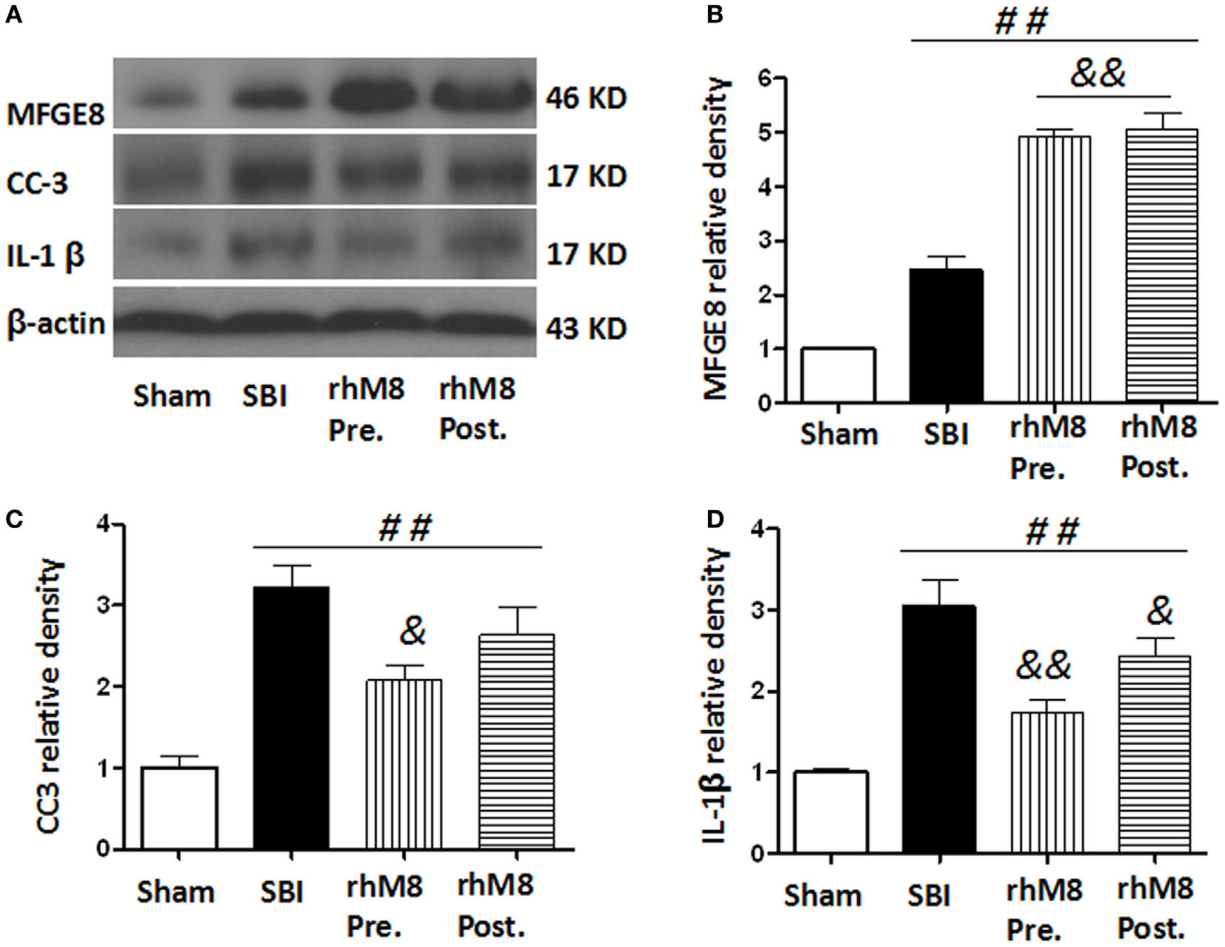
The different effects between recombinant milk fat globule-EGF factor-8 (rhMFGE8) pretreatment (rhM8-pre) and rhMFGE8 posttreatment (rhM8-post) on anti-apoptosis and anti-inflammation at 24 h after surgical brain injury (SBI). Representative blots **(A)** and quantitative analysis of the relative densities of MFGE8 **(B)**, cleaved caspase-3 (CC3) **(C)**, and interleukine-1 beta (IL-1β) **(D)** indicated that the expressions of MFGE8, CC3, and IL-1β were significantly increased at 24 h after SBI. Both rhM8-pre and rhM8-post significantly increased the expression of MFGE8. Meanwhile, only rhM8-pre significantly decreased the level of CC3, but not rhM8-post. In addition, both rhM8-pre and rhM8-post significantly decreased the level of IL-1β, but rhM8-pre had better inhibiting effect. *n* = 6 for each group. ^##^*P* < 0.01 vs. sham group, ^&^*P* < 0.05 vs. SBI group, and ^&&^*P* < 0.01 vs. SBI group.

The treatments all significantly increased the protein levels of MFGE8 (Figures 6A,B, *P* < 0.01), CC3 (Figures 6A,C, *P* < 0.01), and IL-1β (Figures 6A,D, *P* < 0.01) in the SBI model. Administration of rhMFGE8 further increased the protein level of MFGE8 (Figures 6A–C, *P* < 0.01). Pretreatment significantly decreased the expression of CC3 (Figures 6A,C, *P* < 0.05) and IL-1β (Figures 6A,D, *P* < 0.01). However, posttreatment, which only significantly decreased the expression of IL-1β (Figures 6A,D, *P* < 0.05), did not significantly decrease CC3 expression (Figures 6A,C, *P* > 0.05).

### Knockdown of Endogenous MFGE8 Increased the Expression of CC3 and IL-1β at 24 h after SBI

**Figure 7:**
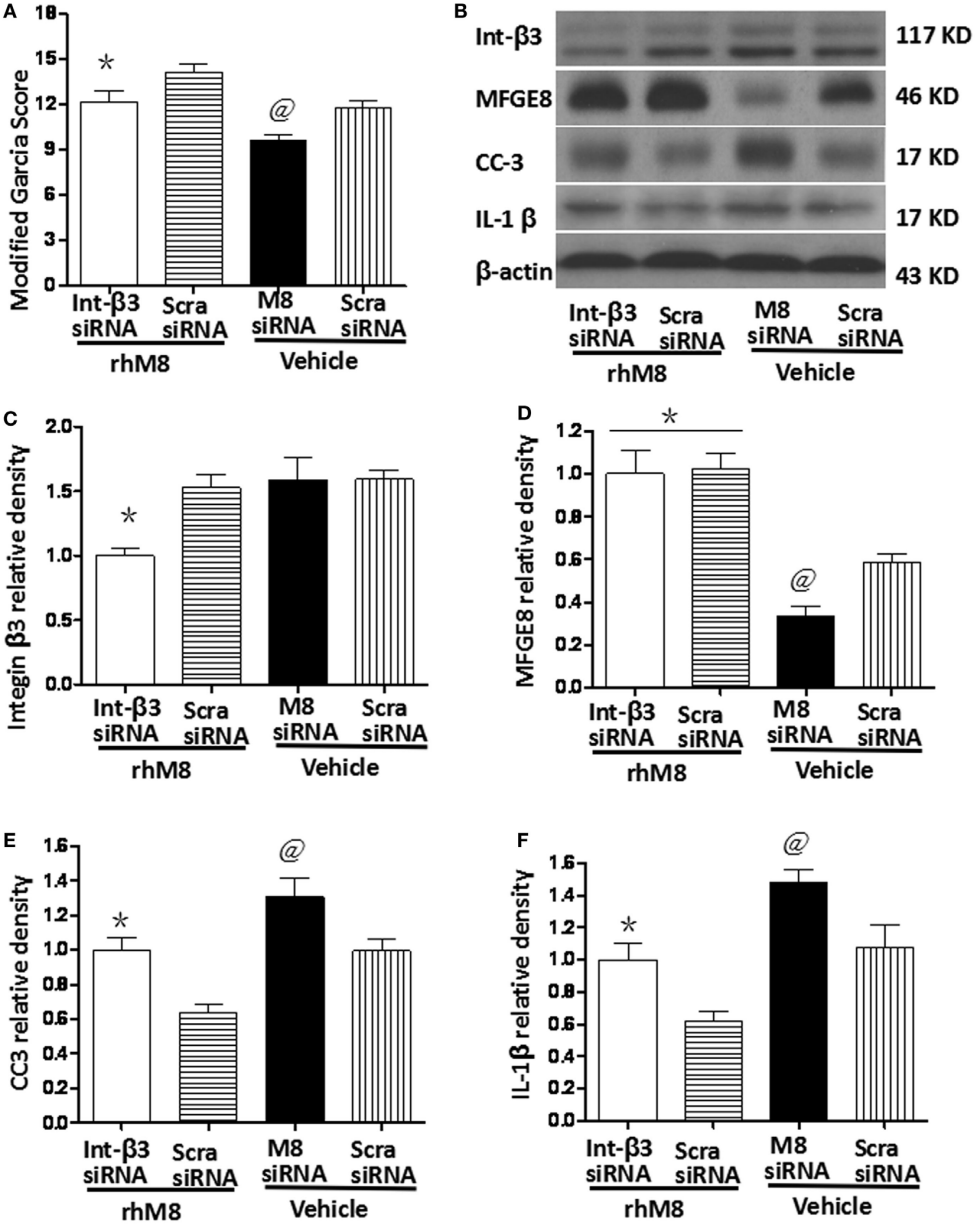
Knockdown endogenous milk fat globule-EGF factor-8 (MFGE8) increased cleaved caspase-3 (CC3) and IL-1 expression. Knockdown integrin-β3 receptor abolished the neuroprotective effects of recombinant milk fat globule-EGF factor-8 (rhMFGE8) treatment at 24 h after surgical brain injury (SBI). **(A)** MFGE8 siRNA significantly deteriorated the modified Garcia score at 24 h after SBI, and integrin-β3 siRNA offset the ameliorating neurological function of rhMFGE8. Representative blots **(B)** and quantitative analysis of integrin β3, MFGE8, CC3, and interleukine-1 beta (IL-1β) **(C–F)** show MFGE8 siRNA significantly decreased MFGE8 expression **(D)** and increased CC3 and IL-1 expression **(E,F)**, and the level of integrin β3 was significantly decreased by integrin-β3 siRNA **(C)** which abolished the effect of MFGE8 decreasing the level of CC3 and IL-1 **(E,F)**. *n* = 6 for each group. *P < 0.05 vs. scramble siRNA + rhMFGE8 (scram siRNA + rhMFGE8) group and ^@^*P* < 0.05 vs. scramble siRNA + vehical (scram siRNA + vehical) group.

Milk fat globule-epidermal growth factor-8 siRNA pretreatment significantly decreased the modified Garcia score at 24 h after SBI (Figure 7A, *P* < 0.05) and knocked down endogenous MFGE8 protein expression (Figures 7B,C, *P* < 0.05), which increased CC6 (Figures 7B,E, *P* < 0.05) and IL-1β (Figures 7B,F, *P* < 0.05) expression. In contrast, scramble siRNA had no significant effects (*P* > 0.05).

### Knockdown of Integrin-β3 Receptor Abolished the Neuroprotective Effects of rhMFGE8 Treatment at 24 h after SBI

Integrin-β3 siRNA blocked the rhMFGE8-mediated increase in Garcia score at 24 h after SBI (*P* < 0.05). Integrin-β3 siRNA efficiently inhibited integrin-β3 protein expression (Figures 7B,C, *P* < 0.05), which has no effects on the protein levels of MFGE8 (Figures 7B,D, *P* > 0.05). Furthermore, integrin-β3 siRNA abolished the rhMFGE8-mediated decrease in expressions of CC3 (Figures 7B,E, *P* < 0.05) and IL-1β (Figures 7B,E, *P* < 0.05) at 24 h after SBI.

## Discussion

In the present study, we investigated the different effects of rhMFGE8 pretreatment and posttreatment and whether the MFGE8/integrin-β3 pathway was involved in the anti-apoptotic and anti-inflammatory effects in a rat model of SBI. Our data showed that rhMFGE8 pretreatment alleviated neurological deficits and decreased brain water content and apoptotic cells in the SBI model, which exhibited neurological dysfunction, apoptosis, and inflammation. Meanwhile, MFGE8 siRNA, which inhibited endogenous MFGE8 expression, significantly increased apoptotic cells and the protein levels of CC3 and IL-1β. Furthermore, knockdown of its receptor integrin β3 by siRNA abolished the effects of rhMFGE8 in the SBI model.

Milk fat globule-epidermal growth factor-8 is a secreted protein for the regulation of apoptotic cells clearance which contains two characteristic functional domains: EGF-like domains, including a RGD-containing sequence that binds to integrin of phagocytic cells, and a blood coagulation factor V/VIII segment binding to phosphatidylserine, which is usually located at inner leaflet of plasma membrane and only exposed in the process of apoptosis (11, 13–15, 26). Therefore, the full engulfment process and apoptotic cells clearance cannot be completed without MFGE8 (27). In the present study, we showed that there were many apoptotic cells surrounding the corticotomy region and increased CC3 protein in the right frontal lobe at 24 h after SBI, which was consistent with previous studies of the SBI model (4). Following administration of rhMFGE8, TUNEL-positive cells were decreased at the periphery of surgical resection, and the CC3 protein level decreased. MFGE8 siRNA inhibited endogenous MFGE8 expression, which increased the expression of CC3 and IL-1β at 24 h after SBI. These results were consistent with previous studies showing that MFGE8 promoted phagocytosis of microglia in an SAH model and that MFGE8 deficiency is detrimental (16). For the engulfment of apoptotic bodies, a specific vitronectin receptor (integrin αvβ3 or integrin αvβ5), which is expressed on phagocytes, binds MFGE8-opsonized apoptotic cells (28, 29). In the present study, knockdown of integrin β3 by siRNA abolished the anti-inflammatory and anti-apoptotic effects of rhMFGE8 in the SBI model, similar to observations made in SAH (16, 24). These findings indicate that rhMFGE8 enhances apoptotic cells engulfment through the MFGE8/integrin-β3 pathway in the SBI model.

Inflammatory mediators likely play a key role in the traumatic (cortical incision) and ischemic (brain retraction) injury associated with SBI (2–6), similar to other brain injuries (7–9). As a vital process to prevent inflammation, apoptotic cells need to be rapidly and efficiently cleared (26, 27). If apoptotic cells are not cleared by phagocytosis, they will eventually undergo secondary necrosis and stimulate an inflammatory response (30, 31). MFGE8 can promote phagocytosis and inhibit inflammation (16). Our data showed that the expression of CC3 was significantly increased 3 h after SBI, but the increase in MFGE8 protein level was not significant. Similar to the SAH model (16), endogenous MFGE8 protein expression began to increase early after the injury, which was not sufficient, and thus, phagocytosis was limited. Only a portion of the phagocytic cells were cleared, and most apoptotic cells showed secondary necrosis, in which the release of dangerous contents led to increased IL-1β protein level, as shown in our results. The potential damage can be alleviated by exogenous MFGE8, which mediated the removal of apoptotic bodies *via* its affinity to phosphatidylserine and decreased inflammation. In the present study, pretreatment with rhMFGE8, which remedied the shortage of exogenous MFGE8, significantly decreased IL-1 protein level and brain water content and significantly alleviated neurological deficits in the SBI model. If administration of rhMFGE8 occurred after the injury, the apoptotic cells were not cleared rapidly, and secondary necrosis and inflammatory responses were observed. Meanwhile, endogenous MFGE8 protein was increased at later time periods, and exogenous MFGE8 was not as important as it was before the injury. Thus, rhMFGE8 posttreatment had poor therapeutic efficacy in the present study. The time point of administering rhMFGE8 influenced its effects, which may explain the dual effects of MFGE8 in different studies.

In addition, literature reports have shown that MFGE8 has anti-inflammatory activity through the activated Nrf2/HO-1 pathway (16) and inhibiting neutrophil migration *via* integrin-αvβ3-dependent MAP kinase activation (32). Meanwhile, the deceased inflammation, which was inhibited predominantly by clearance of apoptotic cells, had anti-apoptosis effects (11, 33–35).

However, in real patients, the outcome after craniotomy is usually assessed weeks to months later. There might be less apoptosis initially when using MFGE8, but eventually all groups (treated and non-treated) might have similar neurological outcomes. Therefore, the long-term assessment after MFGE8 treatment should be evaluated in the further study. In addition, if the craniotomy is being performed to treat cancer, MFGE8 (which decreases apoptosis) might not be favorable. Thus, MFGE8 pretreatment may only suitable for the craniotomy for the patients except brain tumor excision, but further experiments should be performed to observe the effects of MFGE8 for the brain tumor cells.

In the current study, we found that rhMFGE8 pretreatment effectively alleviated neurological deficits and decreased brain water content and apoptotic cells in the SBI model through the MFGE8/integrin-β3 pathway, and treatment time was an important factor in achieving curative effects. Clinical, stroke and trauma patients cannot be cured with pretreatment, but SBI patients can. MFGE8 pretreatment may serve as a promising therapeutic strategy for SBI patients.

## Ethics Statement

All experimental protocols of this study were approved by the Ethics Committee of Central South University and performed according to the eighth edition of the National Institutes of Health Guide for the Care and Use of Laboratory Animals.

## Author Contributions

FL, HF, and JZ conceived and designed the experiments. YX, GL, YZ, KR, and TH performed the experiments. YX, GL, and FL analyzed the data. FL and YC wrote and revised the manuscript. FL gave the final approval for the manuscript to be published.

## Conflict of Interest Statement

The authors declare that the research was conducted in the absence of any commercial or financial relationships that could be construed as a potential conflict of interest.
